# Association between Time of Pay-for-Performance for Patients and Community Health Services Use by Chronic Patients

**DOI:** 10.1371/journal.pone.0089793

**Published:** 2014-02-28

**Authors:** Xi Sun, Zhanchun Feng, Ping Zhang, Xingliang Shen, Li Wei, Miaomiao Tian

**Affiliations:** 1 Medicine and Health Management School, Huazhong University of Science and Technology, Wuhan, Hubei, China; 2 Health Bureau of Jiulongpo, Chongqing, China; 3 Wuhan Union Hospital, Wuhan, Hubei, China; 4 Institute of Medical Information, Center for Health Policy and Management, Chinese Academy of Medical Sciences, Beijing, China; Groningen Research Institute of Pharmacy, Netherlands

## Abstract

**Background:**

Pay-for-performance for patients is a cost-effective means of improving health behaviours. This study examined the association between the pay time for performance for patients and CHS use by chronic patients.

**Methods:**

A cross-sectional study was undertaken to estimate distribution characteristics of CHS use in 2011 and collect data of socio-demographic characteristics (sex, age, education level, occupation, disposable personal income in 2011, distance between home and community health agency), chronic disease number, and time of pay-for-performance for patients. Participants were 889 rural adults with hypertension or type II diabetes aged 35 and above. Standardized CHS use means chronic patients use CHS at least once per quarter.

**Results:**

Patients who received incentives prior to services had 2.724 times greater odds of using standardized CHS than those who received incentives after services (95%CI, 1.986–3.736, P<0.001). For all subgroups (socio-demographic characteristics and chronic disease number), patients who received incentives prior to services were more likely to use standardized CHS than those receiving incentives after services.

**Conclusions:**

Pay time for performance for patients was associated with CHS use by chronic patients. Patients receiving incentive prior to services were more likely to use standardized CHS. And pay time should not be ignored when the policy on pay-for-performance for patients is designed.

## Introduction

Pay-for-performance for patients is a cost-effective means of improving health behaviours [1]. Healthy behaviours can be divided into two categories, simple and complex. Simple behaviours include immunization and follow-up; complicated behaviours include weight loss and smoking cessation [2]. Relatively small incentives could be useful for simple behaviours [3], [4], [5],but big incentives should be preferred for complicated behaviours [6], [7]. In addition, the role of incentives could be positive or negative [8]. According to economic theories, the effects of incentive on behaviours are not only economic drive (causing direct effects) but also motivations upon the subject (causing indirect effects) [9]. Many previous studies have only focused on the relationship between direct effects [1], [10] and behaviours change, such as the form and value of incentives on behaviours; the effect of pay time still requires further investigation and strict examination.

Chronic disease is a major cause of mortality in China [11], [12]. However, sufferers of chronic disease are showing evidence of the so-called three low indexes: low awareness rate of chronic disease knowledge, low treatment rate and low control rate [13], [14]. It is thought that instruction about healthy behaviours will enhance knowledge of chronic disease and reduce the risk of such diseases [15], [16], [17]. And the community health services (CHS) provided for sufferers of chronic disease are very helpful and quite important [15]. According to the new health reform launched in 2009 in China, community health agencies are required to make a joint effort with residents' committees to provide CHS, which include chronic disease examination, blood pressure and blood sugar measurement, instructions for taking medication and maintaining a healthy lifestyle as well as chronic disease knowledge. If a chronic patient uses all these services at least once per quarter, it is called that he or she uses standardized CHS.

This paper is dedicated to conducting a cross-sectional study to estimate the association between the pay time and CHS use by chronic patients.

## Methods

### Study population

Hangu Town locates in western part of Jiulongo District (Chongqing City) and has 30 square kilometres and 20 000 residents. In 2011, five community health agencies in Hangu encouraged community residents to use CHS by paying them washing powder and a towel worth 10 Yuan. Residents in three communities received incentives after using standardized CHS (we called it Type A). Residents in the other two communities received incentives before using CHS (we called it Type B). There were 1356 chronically ill adults in Hangu Town by the end of 2011. The eligible candidates were defined as those who had been diagnosed with hypertension or type II diabetes by physicians, and aged older than 35years. Individuals were not included in the study if they were migrant worker (migrant worker means the rural people leave their villages and go into the cities to do an off-farm work, 459 in total) or if they suffered with mental illness (8 in total). In total, 899 participants were included in in this study. 456 patients were from three communities (patients in these communities would receive washing powder and a towel if they used standardized CHS). 433 patients were from the other two communities (patients in these communities had received the same award before they used CHS).

Socio-demographic information collected included sex, age, educational level, occupation, disposal personal income in 2011(self-reported by patients), distance between home and community health agency. And chronic disease number was also collected. All these information were collected through a questionnaire survey conducted in January 2012. CHS use information was collected from health records.

All participants were interviewed in person, and the study purpose was explained to them by the interviewers. Students from the School of Medicine and Health Management of Tongji Medical College were recruited and trained as interviewers.

This study was approved by the Ethics Committee of Tongji Medical College, Huazhong University of Science and Technology and all participants provided written informed consent in the study.

### Data Analysis

Socio-demographic characteristics (sex, age(35–44, 45–54, 55–64, 65–74 and ≥75 years old), education level (elementary school and below, middle school and high school and above), occupation (farmer, factory workers, government-employed, retired and other), disposal personal income (<10000, <15000, <15000 and ≥20000 Yuan)), distance between home and the community health agency (<1, <2, <3, <4 and ≥4 km), chronic disease number (1 and ≥2)) and pay time (type A and type B) were summarized using descriptive statistics. Prior to developing logistic regression analysis of the predictors of standardized CHS use, Chi-square and Fisher's exact tests (where appropriate) were used to explore differences in values of the covariates for the purpose of selecting meaningful variables for regression. Binary logistic regression analysis was used to model the probability of participants using standardized CHS. The dependent variable was whether (1) or not (0) each participant used standardized CHS. Independent variables included sex, age, educational level, disposal personal income, distance between home and community health agency, chronic disease number and pay time.

In addition, the homogeneity of the association (i.e., interaction) between the group assignment and utilisation rate across the subgroups was assessed with the use of the Cochran and Mantel-Haenszel test. Within each subgroup, the utilisation rates in the two pay time groups were compared with the use of chi-square tests.

All statistical analyses were performed using PASW statistics 12.0 (SPSS, Chicago, IL, USA) with 2-sided statistical tests at a 0.05 significance level.

## Results

The soci-demographic characteristics, chronic disease number and pay time were significantly different between groups of who used standardized CHS and who did not (Table 1).

**Table 1:** Univariate analyses examining factors associated with standardized CHS use in rural adults with chronic disease aged 35 and above.

Predictor		In total (n = 889)		Group of who didn't use standardized CHS (n = 463)		Group of who used standardized CHS (n = 426)		P
		N	%	N	%	N	%	
Sex	Female	482	54.2	207	44.7	275	64.6	<**0.001**
	Male	407	45.8	256	55.3	151	35.4	
Age (yr)	35–44	68	7.6	44	9.5	24	5.6	<**0.001**
	45–54	439	49.4	237	51.2	202	47.4	
	55–64	240	27.0	128	27.6	112	26.3	
	65–74	94	10.6	44	9.5	50	11.7	
	75–	48	5.4	10	2.2	38	8.9	
Educational level	Elementary school and below	470	52.9	300	64.8	170	39.9	<**0.001**
	Middle school	302	34.0	144	31.1	158	37.1	
	High school and above	117	13.2	19	4.1	98	23.0	
Occupation	Farmer	102	11.5	38	8.2	64	15.0	<**0.001**
	Factory employee	304	34.2	194	41.9	110	25.8	
	Government employee	114	12.8	33	7.1	81	19.0	
	Retired	207	23.3	108	23.3	99	23.2	
	Other	162	18.2	90	19.4	72	16.9	
Distance between home and community health agency (kilometers)	<1	174	19.6	79	17.1	95	22.3	<**0.001**
	<2	286	32.2	130	28.1	156	36.6	
	<3	178	20.0	116	25.1	62	14.6	
	<4	203	22.8	130	28.1	73	17.1	
	≥5	48	5.4	8	1.7	40	9.4	
Disposal personal income (Yuan)^a^	<10000	58	6.5	43	9.3	15	3.5	<**0.001**
	<15000	126	14.2	81	17.5	45	10.6	
	<20000	534	60.1	286	61.8	248	58.2	
	≥20000	171	19.2	53	11.4	118	27.7	
Chronic disease number	1	652	73.3	374	80.8	278	65.3	<**0.001**
	≥2	237	26.7	89	19.2	148	34.7	
Pay time	Type A	456	51.3	284	61.3	172	40.4	<**0.001**
	Type B	433	48.7	179	38.7	254	59.6	

Abbreviation: CHS, community health services; N, number of cases; P, P value.

a disposal personal income were self-reported.

Female patients were more likely to use standardized CHS than male patients (95%CI, 0.144–0.946, P = 0.038); patients who were older, or with higher education level, or with higher disposal personal income were more likely to use standardized CHS; patients who received incentives prior to services had 2.724 times greater odds of using standardized CHS than those receiving incentives after services (95%CI, 1.986–3.736, P<0.001) (Table 2).

**Table 2:** Multivariable analyses examining factors associated with standardized CHS use in rural adults with chronic disease aged 35 and above.

Predictor	Reference category	B	P	Odds Ratio (95% CI)
Sex	Female			
Male		−0.998	**0.038**	**0.369 (0.144-0.946)**
Age (yr)	35–44			
45–54		0.989	**0.004**	**2.688 (1.363–5.300)**
55–64		1.46	**0.000**	**4.304 (1.996–9.278)**
65–74		1.531	**0.002**	**4.624 (1.784–11.985)**
75-		2.416	**0.000**	**11.196 (3.406–36.796)**
Educational level	Elementary school and below			
Middle school		0.855	**0.000**	**2.352 (1.484–3.729)**
High school and above		1.377	**0.005**	**3.963 (1.525–10.302)**
Occupation	Farmer			
Factory employee		−0.728	0.155	0.483 (0.177–1.318)
Government employee		0.13	0.819	1.139 (0.375–3.460)
Retired		−0.501	0.213	0.606 (0.276–1.334)
Other		−0.316	0.409	0.729 (0.345–1.543)
Distance between home and community health agency (kilometers)	<1			
<2		−0.189	0.532	0.828 (0.458–1.498)
<3		−0.379	0.446	0.684 (0.258–1.814)
<4		−0.437	0.483	0.646 (0.190–2.193)
≥5		0.538	0.541	1.713 (0.304–9.644)
Disposal personal income (Yuan)^a^	<10000			
<15000		0.532	0.209	1.703 (0.743–3.906)
<20000		1.604	**0.000**	**4.975 (2.153–11.498)**
≥20000		1.838	**0.000**	**6.283 (2.428–16.262)**
Chronic disease number	1			
≥2		0.505	0.118	1.657 (0.880–3.123)
Pay time	Type A			
Type B		1.002	**0.000**	**2.724 (1.986–3.736)**

Abbreviation: CHS, community health services; B, partial regression coefficient; P, P value.

a disposal personal income were self-reported.

Table 3 shows the utilisation rates stratified according to subgroup. For all subgroups, members of pay time Type B had higher utilisation rates than members of pay time Type A. None of the tests for interaction showed significant differences in this population; instead, the observed patterns showed a consistent effect of pay time across numerous characteristics.

**Table 3:** Standardized CHS use rates of pay time type A and B in various subgroups.

Characteristics	Type A	Type B	Odds Ratio (95% CI)
	*no.who used standardized CHS/Total no.*		
Sex			
Male	63/216	88/191	**2.07 (1.38–3.12)**
Female	109/240	166/242	**2.63 (1.81–3.81)**
Age (yr)			
35–44	9/36	15/32	2.65 (0.95–7.38)
45–54	84/224	118/215	**2.03 (1.38–2.97)**
55–64	45/126	67/114	**2.57 (1.52–4.32)**
65–74	18/48	32/46	**3.81 (1.62–8.98)**
75-	16/22	22/26	2.06 (0.50–8.53)
Educational level			
Elementary school and below	68/247	102/223	**2.22 (1.51–3.26)**
Middle school	59/155	99/147	**3.36 (2.09–5.39)**
High school and above	45/54	53/63	1.06 (0.40–2.84)
Occupation			
Farmer	25/51	39/51	**3.38 (1.45–7.90)**
Factory employee	38/144	72/160	**2.28 (1.41–3.70)**
Government employee	48/68	33/46	1.06 (0.46–2.42)
Retired	36/105	63/102	**3.10 (1.76–5.46)**
Other	25/88	47/74	**4.39 (2.26–8.51)**
Distance between home and community health agency (kilometers)			
<1	34/87	61/87	**3.66 (1.95–6.86)**
<2	65/146	91/140	**2.31 (1.44–3.73)**
<3	24/94	38/84	**2.41 (1.28–4.53)**
<4	31/108	42/95	**1.97 (1.10–3.52)**
≥4	18/21	22/27	0.73 (0.15–3.49)
Disposal personal income (Yuan)^a^			
<10000	6/29	9/29	1.73 (0.52–5.69)
<15000	12/69	33/57	**6.53 (2.89–14.75)**
<20000	105/276	143/258	**2.03 (1.43–2.86)**
≥20000	49/82	69/89	**2.32 (1.19–4.52)**
Chronic disease number			
1	118/343	160/309	**2.05 (1.49–2.81)**
≥2	54/113	94/124	**3.42 (1.97–5.95)**

Abbreviation: CHS, community health services.

a disposal personal income were self-reported.

## Discussion

According to this paper, the subjects of Type A received washing powder and a towel worth 10 Yuan as an award after engaging with services. Those of Type B, however, received the same award before partaking in these services. The results showed that the utilisation rate of the standardised CHS of Type B was much higher than that of Type A. Why would this be the case?

One possible reason might be that different time of incentives lead to differential effects on human psychology, bringing about different behaviours. According to behavioural economics, economic incentives can not only motivate humans economically, but also lead to indirect effects on human psychology [9]. The indirect effects include the crowding-out effect [18], [19]. This effect has two aspects. The first is the so-called principal-agent relationship in which the incentive will be seen as a sign of distrust by the agent, which lowers motivation. The incentive might also been as a compromise to personal reputation, which also lowers motivation. The second is an initial promotion of motivation [20]. It is obvious that the subjects of Type A and B were subject to the incentive both psychologically and behaviourally. However, Type B subjects were given the incentive prior to engaging in services so that they sensed adequate trust. According to the principal-agent relationship, a high level of confidence by encourages them to achieve the task entrusted to them more effectively [21].

Moreover, considering the tendency of holding optimistic attitudes toward the future [22], all of the subjects of Type B accepted the award and possibly promised to themselves (which is seen as self-comfort or a positive psychological suggestion) that they would engage in the standardised community-based health management services. This promise of positive psychological suggestion affected their behaviours.

## Limitations

First of all, some other predictors affecting the standardized CHS use by chronic patients aged 35 and above may be omitted.

Second, although the community health agency that was the sample source indicated that overall examination of residents with chronic disease older than 35 years had been fully implemented, the chance of omission, which could result in selection bias, remains.

In addition, based on behavioural economics, the conditions needed to make a change in simple behaviours and complicated behaviours are quite different [1]. Given that making a change in complicated behaviours involves more energy and time, as well as often involves the sacrifice of comfortable experiences in lifestyle, long-term change might also be expected to be more difficult to change with such an award in this study. This paper only focuses on relatively simple behaviours. The association between pay time for performance for patients with complicated behaviours change still requires further research.

## Conclusions

To conclude, this paper has found that pay time for performance for patients was associated with CHS use by chronic patients. Patients receiving incentive prior to services were more likely to use standardized CHS. And pay time should not be ignored when the policy on pay-for-performance for patients is designed.
